# Diet and Acanthosis Nigricans over a Two-Year Period in Children of the Pacific Region

**DOI:** 10.3390/nu15122718

**Published:** 2023-06-12

**Authors:** Douglas Taren, Halimatou Alaofè, Ashley B. Yamanaka, Patricia Coleman, Travis Fleming, Tanisha Aflague, Leslie Shallcross, Lynne Wilkens, Rachel Novotny

**Affiliations:** 1 Section of Nutrition, Department of Pediatrics, School of Medicine, University of Colorado, Aurora, CO 80045, USA; 2 Health Promotion Sciences, Mel and Enid Zuckerman College of Public Health, University of Arizona, Tucson, AZ 85724, USA; halaofe@arizona.edu; 3 Human Nutrition, Food and Animal Sciences Department, College of Tropical Agriculture and Human Resources, University of Hawai’i at Mānoa, Honolulu, HI 96822, USAnovotny@hawaii.edu (R.N.); 4 Cooperative Research, Extension, and Education Service, Northern Marianas College, Saipan, MP 96950, USA; 5 Agriculture, Community and Natural Resources Division, Samoa Community College, Pago Pago, AS 96799, USA; 6 Cooperative Extension and Outreach, College of Natural and Applied Sciences, University of Guam, Mangilao, GU 96913, USA; 7 Health, Home and Family Development, UAF Institute of Agriculture, Natural Resources and Extension, University of Alaska Fairbanks, Fairbanks, AK 99775, USA; 8 Biostatistics Shared Resource, University of Hawai’i Cancer Center, University of Hawai’i at Mānoa, Honolulu, HI 96813, USA

**Keywords:** type 2 diabetes, acanthosis nigricans, sugar, carbohydrate, diet, body mass index

## Abstract

Background: The impact that dietary carbohydrates have on children developing type 2 diabetes remains controversial. Furthermore, there are limited pediatric longitudinal studies on changes in body mass index (BMI) and diet related to the development of acanthosis nigricans (AN), a risk factor associated with type 2 diabetes. Methods: Two 24 h dietary records were collected for 558 children, 2–8 years of age, at baseline and at a 2-year follow-up. Data on age, sex, BMI, and the presence of AN were also collected at each time point from the Children’s Healthy Living Program. Logistic regression was used to determine factors associated with the presence of AN at follow-up. Multinominal regression was used to determine factors associated with changes in AN status. Linear regression was used to measure the associations between changes in dietary intake and in the Burke Score for AN. Results: AN was present in 28 children at baseline and 34 children at follow-up. Adjusting for the presence of AN at baseline, age, sex, study group, baseline BMI, change in BMI z-score, time between assessments, and baseline intake, an increase from baseline for each teaspoon of sugar and serving of carbohydrate-rich food increased the risk for having AN at follow-up by 9% and 8%, respectively (*p* ≤ 0.05). An increased intake of added sugar (teaspoons) increased the risk of developing AN by 13% (*p* ≤ 0.01) and an increase in servings of foods rich in starch increased the risk of developing AN by 12% (*p* ≤ 0.01) compared to children who never had AN. Increasing the intake of fruit was also associated with decreased Burke Scores using multiple regression. However, the intake of energy and macronutrients were not associated with AN. Conclusions: Added sugar and foods rich in starch were independently associated with the occurrence of AN, suggesting the type of carbohydrates consumed is a factor in AN occurrence.

## 1. Introduction

The prevalence of obesity in children in the United States (USA) and globally has significantly increased, disproportionately for children of color [1,2,3]. An earlier study reported that the prevalence of obesity in this population of 2–8-year old children was 14.0% and greater in boys (16.3%) compared to girls (11.6%), and 16.3% for children 6–8 years of age [4]. Additionally, the presence of acanthosis nigricans was also significantly associated with obesity, with an odds ratio of 9.2 (95% confidence limit of 6.69–12.80). Additionally, children with obesity are at a greater risk for hyperlipidemia, cardiovascular disease, hypertension, type 2 diabetes mellitus (T2D), and associated health care costs [2,5,6,7,8,9,10]. In the United States, it was estimated that health care costs for diabetes were USD 101.4 billion, representing the greatest proportion of the nation’s health care costs [11]. Furthermore, medical care for children with T2D ranged from USD 1798 to USD 2938 per year (2014 USD) depending on the type of treatment provided [12]. In the Pacific region, the prevalence of children with obesity in 2015 increased with age and was identified in 21% and 39% of 2-year-olds and 8-year-olds, respectively [13].

There are several measurements that are used to determine if a person has insulin resistance, poor insulin secretion, and poor glucose control [14]. These include single measurements, such as blood glucose levels and hemoglobin A1c levels, and using fasting insulin and glucose concentrations for the homeostatic model assessment, HOMA-IR [15]. Additionally, acanthosis nigricans (AN) is recognized as a clinical indicator of insulin resistance [16,17,18,19,20,21,22], which often precedes the development of T2D [23,24]. AN is defined by hyperkeratosis, which can include a dark, velvety discoloration of the skin that is present in the flexural areas, such as the axillae, groins, behind the knees, and the neck, and is associated with HOMA-IR and serum adiponectin levels [25]. The Endocrine Society recommends conducting an assessment for the presence of AN when suspecting insulin resistance in children and recommends against measuring insulin concentrations when evaluating children or adolescents for obesity, [5] and the American Diabetes Association has included AN as an adolescent risk factor for diabetes. The Burke Score for the size of an AN lesion has been associated with the severity of insulin resistance [26,27].

Screening school-aged children for AN has also been recommended [28]. Identifying predictors associated with preventing and decreasing AN can provide evidence-based guidance to caregivers and decrease the medical and economic burden of living with T2D. Although body mass index (BMI) has been associated with the presence of AN, there have been few longitudinal studies in young childhood (e.g., <10 years of age) on dietary factors associated with AN. Furthermore, the role that dietary sugars and foods high in starch have on the development of T2D remains controversial [29,30,31,32].

The Children’s Healthy Living (CHL) Program was a community randomized cluster environmental intervention trial to modify community and individual risk factors to prevent young childhood obesity [33,34,35]. We report findings from a two-year longitudinal subset to determine if nutritional factors are associated with AN. We hypothesized that AN would be associated with changes in a child’s BMI z-score and diet, specifically that an increased intake of added dietary sugar and foods high in starch would increase the risk of having AN.

## 2. Materials and Methods

### 2.1. Research Setting

The CHL program was conducted in the following USA Affiliated Pacific region jurisdictions: the USA territories of American Samoa, the Commonwealth of the Northern Mariana Islands, Guam, and the US states of Hawai’i and Alaska. The communities in each jurisdiction selected for inclusion in the CHL met the following criteria: sufficient size (>1000 individuals) based on the 2010 jurisdiction censuses, sufficient representation of individuals of indigenous descent, sufficient representation of children, and reasonable accessibility to the CHL team. A complete description of how these communities were selected is available [35,36,37]. Twenty-seven communities, 4–6 per jurisdiction, were included, with 9 randomized to the CHL intervention, 9 to the control condition, and 9 included as temporal communities. The trial included children 2–8 years of age in a baseline cross-sectional sample and a 24-month follow-up cross-sectional sample, expanding the age to 10 years old to measure the impact of the intervention program. 

An initial sample of 703 children were eligible for this study. However, only children who had their weight and height measured for BMI, provided two 24 h dietary recalls at baseline and follow-up, and were assessed for AN at baseline and follow-up were included in this analysis (Supplementary Figure S1). The final study sample included 558 children. There was no significant difference between the children with complete data and those who had a 24 h recall missing in terms of the percentage who had AN: 5.0% vs. 4.4%, respectively. There was also no statistically significant difference in the mean age or mean BMI z-scores between the two groups. However, the percentage of girls who had missing data (22.8%) was significantly greater (*p* ≤ 0.05) than the percentage of boys who had missing data (16.3%) and a greater percentage (*p* ≤ 0.01) of children in the control group (24.3%) had missing data at either time point compared to the intervention group (13.5%).

The Institutional Review Boards of the University of Hawai’i at Mānoa, the University of Guam, and the University of Alaska at Fairbanks approved this study protocol. All other participating jurisdictions ceded IRB approval to the University of Hawai’i at Mānoa. The primary trial was registered at clinicaltrials.gov, #NCT01881373. The experimental design complied with the ethical principles laid out in the 2008 revision of the Declaration of Helsinki and was approved by an ethics committee prior to the study launch (No. NCT04041934). Parents consented, and children ages 6 years and older assented to participate. 

### 2.2. Data Collection

The primary data for this analysis consisted of demographic, anthropometric, and dietary data, along with the presence or absence of AN at baseline and follow-up [34,38,39,40,41,42]. Anthropometric measures were conducted by trained enumerators. Weight and height values were used to calculate the BMI z-score for age and sex based on the USA Centers for Disease Control and Prevention reference standards [43,44]. Each site used the same equipment for taking these measurements (Perspective Enterprises Stadiometer model PE-AIM-101, Seca scale Model 876 (Seca, North America West Office, Chino. CA, USA). All anthropometric measures were standardized to a single expert with intra-measurer reliability of 0.999 for both weight and height [39]. Three measurements were taken for each anthropometric feature; if 2 of the 3 were not within 0.2 units, the first 3 measurements were discarded and an additional set of 3 measurements was taken. The three measurements were averaged for analysis. The SAS program (SAS Institute Cary, NC, USA) for calculating the BMI z-scores was used to classify children with a BMI z-score ≥ the 85th and <95th BMI percentiles as being overweight, and those with a BMI z-score ≥ 95 were classified as having obesity [45,46].

Dietary data were collected using food intake records for two randomly assigned non-consecutive days at each time period by a surrogate (parent/caregiver) to assess the energy, nutrient, and food group intakes of the children. The nutrient and food group data were adjusted using the Statistical Program to Assess Dietary Exposure (SPADE) method to remove day-to-day variability using estimates of within-person and between-person variances [42,47]. The resulting daily values required more than one day of intake per time period and were averaged across days, weighting weekdays as 5/7 and weekend days as 2/7 to better reflect the habitual diet [42,47]. All parents/caregivers were provided with a tool kit of utensils to estimate portion sizes and Ziploc^®^ bags (SCJohnson, Racine, WI, USA) so they could include food labels used to help code foods consumed. The research staff reviewed the dietary records for completeness, and the accuracy of the recorded data with the parents/caregivers after the second day of food intake was recorded. The Pacific Tracker 3 (PacTrac3), developed by the CHL, was used to analyze the food intake (https://nappactrac31.ctahr.hawaii.edu/default.htm, accessed on 28 June 2021); the associated food composition database is maintained by the Nutrition Support Shared Resource at the University of Hawai’i Cancer Center [40]. 

The presence of AN on the back of the neck was determined by trained enumerators using the method described by Burke et al. [26]. The AN scores were recorded from zero for no visible AN on close inspection to four for a more prominent presence of AN, which includes a darkening of the skin that extends anteriorly and is visible from the front. We also identified children who did not have AN at either baseline or follow-up as the “Never Group”; those who had AN at both time periods as the “Chronic Group”; those who did not have AN at baseline but had AN at follow-up as the “Developed Group”; and those who had AN at baseline but not at follow-up as the “Remission Group.”

### 2.3. Data Management and Analysis

The data validation steps that occurred to prepare a final analysis data set for the CHL have been previously described [33,39,41,48]. We calculated the intake for energy (kcals), added energy from fat and sugar, and the intake of macronutrients (g) and water. The food groups and serving sizes studied for this analysis were based on the Healthy Eating Index (HEI) at the time of the project (HEI-2005) [49]. We also calculated the total number of servings from all animal-sourced foods, the amount of sugar added to food (tsp), and the grams of sugar-sweetened beverages (SSBs) consumed. We calculated the number of servings of starch-rich foods as the sum of teaspoons of added sugar and servings of non-whole grain (mostly rice and white bread), potato, and starchy vegetables (e.g., taro, cassava, yam). Added sugar and refined grains contributed 63% and 36%, respectively, to the total number of servings of starch-rich foods at both baseline and follow-up. The “added sugar” component was based on the “empty calories” HEI-2005 category. A preliminary analysis was completed with 318 children in the CHL study in order to put into context the dietary patterns in the Pacific region, and it was found that the top three SSBs were (1) tea, sweetened; (2) fruit punch drinks; and (3) soda, and they accounted for 47% of the reported SSB intake [50].

### 2.4. Statistical Approach and Power: Post-Hoc Analysis

Prior to the start of this secondary analysis, the research team develop an a priori hypothesis that dietary sugar and foods high in starch would be associated with the presence of AN. Although the focus of this study was on carbohydrates, we also measured the association between 24 dietary components and the presence of AN without any adjustment for multiple testing. The statistical significance of values between the groups was set at *p* ≤ 0.05. Baseline values and changes in the nutritional values between follow-up and baseline were calculated, and Student’s *t*-test was used to measure differences between children with and without AN at follow-up.

Multiple logistic regression was conducted to determine how changes in dietary intake over the two-year period were associated with the presence of AN at follow-up. All models were adjusted for age, sex, experimental group (intervention versus control), baseline BMI, change in BMI z-score over two years, time between assessments, and baseline nutrient or food group intake. Independent models were calculated for each nutrient or food group, and adjusted odds ratios (ORs) with 95% confidence limits were calculated separately for each model. 

Multinominal odds ratios (MORs) were calculated using multinominal regression [51] to determine factors associated with changes in AN status over the two years as classified by Law et al. [52]. Four groups of children were identified: (1) AN not present at both periods (Never), (2) AN present at baseline and follow-up (Chronic), (3) AN present only at follow-up (Developed), and (4) AN present only at baseline and not at follow-up (Remission). Each group was compared with the Never group, and each model that measured the association with a nutrient or food group was adjusted for age, sex, experimental group, baseline BMI, change in BMI z-score over two years, time between assessments, and baseline nutrient or food group intake. Independent models were used to calculate the MORs and 95% confidence limits of each nutrient or food group.

Separate linear multiple regression models were used to determine which dietary measures related to changes in the Burke Score for AN over the two-year period. Linear models were adjusted for age, sex, experimental group, baseline BMI, change in BMI z-score over two years, the initial Burke Score, time between assessments, and baseline nutrient or food group intake.

## 3. Results

### 3.1. Study Population

The average age was 64.6 ± 21.4 (SD) months at baseline; 4.9% had AN at baseline and 5.6% had AN at follow-up. The average time between measures was 24.5 ± 4.5 months. The children were diverse in terms of race, with 67.2% being Native Hawaiian or Pacific Islanders, 16.8% having more than one race, 10.6% Asian, 5.5% White, and 0.4% American Indian or Alaskan Native. At baseline, 5.0% of children presented with AN, 34.6% had a BMI z-score greater than the 85th percentile. At baseline, there was a significant difference (*p* ≤ 0.05) in the percentage of boys who had AN (6.7%) compared to girls (3.1%). The mean age of children with AN was 68.6 months, compared to 64.4 months for children without AN; this was not statistically different. A significantly (*p* ≤ 0.001) greater percentage of children with a BMI z-score above the 85th percentile had AN (11.4%) compared to children who had a lower BMI z-score (1.6%).

Children who had AN at follow-up were significantly (*p* ≤ 0.001) older, had a greater mean BMI z-score, and were more likely to have a BMI z-score ≥ the 85th percentile than children without AN (Table 1).

### 3.2. Predictors of AN at Follow-Up

The children with AN at follow-up had a significantly greater baseline dietary intake of protein and higher numbers of servings of meat, animal-sourced food, potato, vegetables, and grams of water compared to children without AN (Table 2). Children with AN at follow-up significantly increased their intake of the amount of added sugar and the total number of servings of foods rich in starch compared to children without AN at follow-up, but there was also a significant decrease in potato intake of children with AN compared to children without AN at follow-up (Table 3).

Each teaspoon of added sugar and serving of carbohydrate-rich foods was associated with increases of 9% and 7%, respectively, in having AN at follow-up, after adjusting for baseline intake, sex, age, and the baseline and change in BMI values (Table 4). An increased intake of citrus from baseline to follow-up, as well as total fruit intake, was associated with a decreased risk for AN at follow-up. Changes in intake of the other food groups and macronutrients were not associated with having AN at follow-up. 

### 3.3. Predictors of Change in AN Status

A total of 508 of the children (91.0%) never had AN at baseline or follow-up (Never group). Of the children who had AN at baseline, 57.1% (16/28) did not have it two years later (Remission group), while 42.8% (12/28) continued to have AN (Chronic group). Twenty-two of the 530 children (4.2%) developed AN by the 2-year follow-up (Developed group).

Only 1 of the 12 children in the Chronic group had an improvement in their Burke Score by 1 unit; 7 children had no change in their score, and 4 had an increase in their score by 1 unit. Of the 22 children who developed AN, 10 had a score of 1, 5 had a score of 2, 5 had a score of 3, and 2 had a score of 4 at follow-up. Of the 16 children who initially had AN but not at follow-up (score of zero), 11 had a score of 1 at baseline, 3 had a score of 2, and 2 had a score of 3.

The Chronic group had the greatest mean baseline BMI z-score, followed by children who developed AN, and children who never had AN had the lowest mean BMI z-scores (Table 5). Nearly all (92.3%) of the children in the Chronic group had a BMI z-score greater than the 95th percentile at baseline, while it was slightly lower for those in the Developed group (79.2%) and lowest for those who were in the Remission group (47.4%). Fewer children (12.4%) had a BMI z-score at baseline equal to or greater than the 95th percentile who were in the Never group. The 2-year changes in the mean BMI z-scores were different across the four AN groups but were not statistically significant. However, the Developed group had the greatest 2-year increase in their mean BMI z-scores compared to the other groups. Those in the Remission group had the greatest 2-year decrease in their mean BMI z-scores (Table 5).

The 12 children who had AN at both time periods had a BMI z-score ≥ the 85th percentile at both time periods. For the children who developed AN (n = 22), 21 had a BMI z-score ≥ the 85th percentile at baseline and follow-up. For the 16 children who initially had AN at baseline but not at follow-up, 7 had a BMI ≥ the 85th percentile at both time periods, and 9 had a BMI z-score < 85th percentile at follow-up. For children who never had AN, 320 (63%) never had a BMI z-score ≥ the 85th percentile, 118 (23.2%) had a BMI z-score ≥ the 85th percentile at both time periods, 32 (6.3%) had a BMI ≥ the 85th percentile only at baseline, and 38 (7.5%) had a BMI ≥ the 85th percentile only at follow-up.

The reported mean baseline measures of protein, red meat, and animal-sourced foods differed across the four AN groups, with the Chronic group having the greatest intake (*p* ≤ 0.05) and the Never group having the lowest intake (*p* ≤ 0.05) of potato (Supplementary Table S1).

Children who developed AN had the greatest increase across the two-year period in the intake of energy, carbohydrates, protein, added sugar, SSBs, and starch-rich foods. Interestingly, the children in the Chronic group reported a decreased intake of these dietary components (Supplementary Table S2).

An increase in the consumption of added sugar and starch-rich foods was associated with the development of AN compared to those who never had AN after controlling for BMI values and other factors. However, an increase in carbohydrate-rich foods was associated with a decreased risk for the Chronic group compared to the Never group (Table 6).

Multiple regression models resulted in a non-statistically significant association (ß = 0.01, SE = 0.00) between an increase in added sugar and an increase in the Burke Score (*p* ≤ 0.10) when adjusted for age at baseline, time between measurements, baseline BMI z-scores, the change in the BMI z-score, and changes in energy intake and baseline food intake. On the other hand, there were significant associations between an increase in citrus (ß = −0.19, SE = 0.08), other fruit consumption (ß = −0.14, SE = 0.05), and total fruit intake (ß = −0.12, SE = 0.04) and a decrease in the Burke Score (*p* ≤ 0.01). 

## 4. Discussion

The most consistent result of this study is that added sugar and servings of starch-rich foods were associated with an increased risk for AN and a greater increase in the Burke Score. This is also the first and largest longitudinal study, to our knowledge, determining an association between diet and AN in young children after controlling for BMI. The 5% prevalence of AN in this sample is greater than in past studies. Law et al. [52] conducted a 2-year follow-up study with an initial 1% AN prevalence in 4059 children in kindergarten to second grade. Furthermore, the 2-year changes in AN status were more dynamic in the current study, as 8.4% of children changed their status of having or not having AN compared to 2.4% in the study by Law, which represented a cohort of White children in West Virginia. Our study is consistent with previous longitudinal pediatric studies that have consistently reported that the presence of AN is associated with obesity [52,53]. Our current study provides evidence that an increase in the BMI z-score, after adjusting for baseline z-scores, was associated with chronically presenting with AN, while a decrease in the BMI z-score was associated with a remission of AN. Age was an important factor. Children in the Chronic and Developed groups were older, possibly due to adipose rebound, which can occur in children 5–7 years of age [54]. Adiposity rebound has been associated with metabolic changes, including increases in insulin resistance and the concentration of low-density lipoproteins and a decrease in high-density lipoproteins at 12 years of age [55].

Our method of having a proxy report the dietary intake of young children using two days of dietary records is considered one the most accurate methods for collecting dietary intake data of children who have a BMI z-score ≥ 2 [56]. Our current results are even more encouraging in terms of finding an association between sugar intake and foods rich in starch since measuring dietary changes in longitudinal studies can lead to a Hawthorn effect and increase the chance of socially desirable responses, which would bias the results toward the null [57,58]. Thus, it is possible that parents who were informed about the presence of AN in their children and its relationship to diabetes may have under-reported their child’s added sugar intake at follow-up intake due to social desirability given they were participating in a study on childhood obesity [59]. A previous analysis of NHANES data indicated that adult men and women who self-identified with diabetes had 2.1 and 3.9 times greater risk, respectively, for underreporting energy intake [50,60]. Evidence that AN is associated with insulin resistance comes from several studies [25,27,61,62]. Children in Northern India with obesity and AN had greater HOMA-IR scores and lower adiponectin concentrations compared to children with obesity but without AN [25]. Additionally, Burguete-García et al. reported similar results, where Mexican children with a Burke Score of 3 had significantly greater HOMA-IR values than children with lower Burke scores [61]. Nithun et al. also reported that AN and the Burke Scores were also associated with HOMA-IR values in patients 2–24 years of age [27]. Furthermore, Nsiah-Kumi et al. reported that AN was a specific, albeit not sensitive, indicator of insulin resistance in American Indian and Alaska Natives using results from a cross-sectional study of children 5–18 years of age comparing HOMA-IR values between those with and without AN [62]. Additionally, children with AN and a BMI ≥ the 95th percentile were found to be at greater risk for metabolic syndrome compared to children with AN and a lower BMI [63]. It is also possible that our study population allowed us to measure the impact of diet since Asians and Pacific Islanders may be at greater risk for insulin resistance [21,64,65].

The hyperpigmentation of the skin with AN is associated with the insulin-like receptors on keratinocytes and dermal fibroblast, and potentially an estrogen-induced increase in the formation of glycosaminoglycans [66,67,68]. The current results raise the question of how diet and obesity result in AN for some children. Given that refined carbohydrates result in raised post-prandial glucose concentrations, future studies and clinical practices should determine if the frequency and length of spikes in blood glucose concentrations are associated with AN. This approach may assist with providing recommendations to caregivers about the importance of weight management and diet regarding AN and insulin resistance.

This current analysis has some limitations. First, even though, in multiple studies, AN has been associated with insulin resistance, we did not have biochemical indicators of insulin resistance because we did not have human subject approval to collect blood, and it would have been difficult to collect it in the research settings in which this study was conducted. Although this does not allow us to demonstrate a direct association between a decrease in insulin resistance with changes in dietary intake, it is well-established that childhood obesity is associated with insulin resistance [69]. Additionally, AN is a clinical marker of insulin resistance, and the lesion often remains, with weight loss being the most effective way to have it disappear [70]. Thus, finding an association between dietary changes and AN, after adjusting for the presence of obesity, suggests a shift in insulin resistance which, however, requires confirmation with biochemical measures. We also did not use measures of physical activity as an adjustment factor, as it would have substantially reduced our sample sizes. Lower physical activity has been associated in past studies with the presence of AN in older children, but some studies have only been cross-sectional or have not controlled for factors such as diet and BMI [71,72,73,74,75]. A study by Yee et al. used cumulative scores that combined measures of physical activity and diet, which did not make it possible to distinguish the independent impacts of diet and physical activity [76]. Insulin sensitivity may directly increase with physical activity and is also likely to have an effect related to its association with BMI [77,78,79].

Measuring the presence and scoring of AN as a surveillance tool is limited. One limitation of conducting studies on AN is the limited number of AN cases. Screening for AN has been recommended to be part of school-based health screening programs and other surveillance systems that can measure factors associated with AN [28]. For example, Texas has been measuring the presence of AN as part of a health surveillance program in schools and reported that 21,395 children (4.0%) in the first, third, fifth, and seventh grades had AN during the 2020–2021 academic year [80]. However, this program has not used the Burke Score, which may be a more sensitive measure of change over time compared to presence or absence. To determine if the presence of AN and the Burke Score should be used in the surveillance program, additional biochemical validation of its association with dietary markers would be helpful. The cost of surveillance programs could be reduced if AN scores can be used to estimate the amount and severity of insulin resistance, rather than biochemical markers. This would lead to an approach to determine populations at risk and to assess changes in a population’s status. 

As reported in this study of very young children, AN is already becoming apparent in children with obesity. In a review of 25 years of publications, it was reported that there has been an increase in the prevalence of AN in children that parallels the increased prevalence of obesity [81]. However, the presence of AN is not only present in children with T2D. An Italian study reported that the prevalence of AN in 138 children with Type 1 diabetes (T1D) was 5.1%, compared to the 34.6% prevalence rate in a group of 162 obese children [82]. Similarly, a study in India from a tertiary care facility reported that AN was present in 2.6% of patients with T1D who were under 20 years of age, compared to the 47.2% prevalence rate in patients with T2D [83]. Potential reasons for these differences may have been due to the fact that patients with T1D were on medication for glucose control. 

One of the driving forces of the increased prevalence of AN and T2D in children, especially in low- and middle-income countries is the nutrition transition, resulting in a greater prevalence of childhood obesity [84]. This has also led to the double burden of malnutrition in Asia and the Pacific Islands in locations where there is an excessive number of children who are underweight or overweight, including the presence of micronutrient deficiencies [85,86]. Accompanying the increased prevalence of obesity is also a transition in the trade to the Pacific Region that is contributing to a greater availability of low-cost ultra-processed foods that are high in fat and sugar and which are being fed to children [87,88]. These temporal changes have resulted in a low consumption of fruits and vegetables in children in the US Associated Pacific Islands [89]. Additionally, even with an intervention that increased the household consumption of fruit and vegetables over a one-year period in Pohnpei, Federated States of Micronesia, there was an increase in the consumption of soda and imported sweetened foods during the same time period [90].

The results of this study suggest that there is an association between an increased intake of simple carbohydrates and the presence of AN. A recent meta-analysis also provided evidence that increased adiposity is associated with a greater intake of sugars [91]. This new understanding will allow for the formulation of specific advice regarding weight management and diet, which could decrease the rising prevalence of T2D, especially in young Pacific children and other understudied indigenous and minority children at risk of overweight, obesity, and T2D. The results will impact the initiation and design of intervention programs and the modification of existing programs that target young children to decrease the presence of AN and prevent TD2. Additionally, interventions that focus on changing dietary behaviors must also address dietary patterns and the social determinants that are associated with a child’s diet, including socioeconomic status, education, the environment, and food policies.

## 5. Conclusions

The results of this study suggest that there is an association between an increased intake of simple carbohydrates and the presence of AN even after adjusting for BMI. This new understanding will allow for the formulation of specific advice regarding weight management and diet, which could decrease the rising prevalence of T2D, especially in young Pacific children and other understudied indigenous and minority children at risk of overweight, obesity, and T2D. The results will impact the initiation and design of intervention programs and the modification of existing programs that target young children to decrease the presence of AN and prevent TD2.

## Figures and Tables

**Table 1:** Baseline characteristics of study sample by final acanthosis nigricans (AN) status.

Characteristics	AN Absent at Follow-Up (n = 524) $\bar{x}\pm \mathrm{SE}$ ^1^	AN Present at Follow-Up (n = 34) $\bar{x}\pm \mathrm{SE}$
Age (months) ***	63.9 ± 0.9	75.8 ± 3.2
Girls (%) *	47.5	35.3
Boys (%)	52.5	64.7
BMI z-score ***	0.45 ± 0.05	2.37 ± 0.11
% Normal weight status ***	69.5	2.9
% Overweight	16.4	14.7
% Obese ***	14.1	82.4
% Intervention group	47.9	52.9

BMI = body mass index. ^1^ Mean and standard error. Overweight: ≥85th percentile BMI/age. Obesity: ≥95th percentile BMI/age. Significance using a *t*-test: * *p* < 0.05; *** *p* < 0.001.

**Table 2:** Baseline daily dietary intake by final acanthosis nigricans (AN) status.

Dietary Measures	AN Absent at Follow-Up (n = 524) $\bar{x}\pm \mathrm{SE}$ ^1^	AN Present at Follow-Up (n = 34) $\bar{x}\pm \mathrm{SE}$
Energy (kcal)	1884.1 ± 23.2	1967.3± 95.6
Carbohydrate (g)	252.5 ± 3.2	250.0 ± 13.2
Protein (g) **	69.7 ± 0.9	78.4 ± 3.7
Fat (g)	66.6 ± 0.9	71.8 ± 3.7
Added sugar (tsp)	11.6 ± 0.2	9.8 ± 0.8
Added fat (g)	32.6 ± 0.4	35.0 ± 1.9
Milk (servings)	1.2 ± 0.03	1.1 ± 0.11
Total dairy (servings)	1.4 ± 0.03	1.3 ± 0.10
Citrus (servings)	0.3 ± 0.01	0.3 ± 0.04
Other fruit (servings)	0.8 ± 0.02	0.8 ± 0.08
Total fruit (servings)	1.1 ± 0.03	1.1 ± 0.11
Non-whole grain (servings)	6.4 ± 0.09	6.9 ± 0.40
Whole grain (servings)	0.4 ± 0.02	0.4 ± 0.07
Total grains (servings)	7.0 ± 0.10	7.5 ± 0.40
Egg (oz. equivalent)	0.5 ± 0.02	0.5 ± 0.07
Red meat (oz.)	0.8 ± 0.03	1.0 ± 0.13
All meats (oz.) ***	4.7 ± 0.08	5.7 ± 0.33
Animal-sourced food **	7.0 ± 0.2	8.4 ± 0.6
Potato (servings) *	0.09 ± 0.00	0.13 ± 0.02
Starchy veggies (servings)	0.07 ± 0.00	0.10 ± 0.02
Total vegetables (servings) **	0.71 ± 0.02	0.87 ± 0.06
Starch-rich foods (servings)	18.2 ± 0.3	17.0 ± 1.1
Sugar-sweetened beverages (g)	184.8 ± 7.2	163.4 ± 26.1
Water (g) *	355.8 ± 10.6	427.6 ± 52.9

^1^ Mean and standard error. Significance using a *t*-test: * *p* < 0.05; ** *p* < 0.01; *** *p* < 0.001.

**Table 3:** Two-year changes in daily dietary intake measures by final acanthosis nigricans (AN) status.

Change in Dietary Intake Measures	AN Absent at Follow-Up (n = 524) $\bar{x}\pm \mathrm{SE}$ ^1^	AN Present at Follow-Up (n = 34) $\bar{x}\pm \mathrm{SE}$
Energy (kcal)	136.1 ± 25.2	234.0 ± 133.3
Carbohydrate (g)	16.8 ± 3.7	35.6 ± 19.5
Protein (g)	6.1 ± 0.9	6.5 ± 4.5
Fat (g)	4.9 ± 1.0	7.8 ± 5.0
Added sugar (tsp) *	0.55 ± 0.29	3.41 ± 1.35
Added fat (g)	2.9 ± 0.5	3.7 ± 2.8
Milk (servings)	0.05 ± 0.03	0.18 ± 0.14
Dairy (servings)	0.06 ± 0.04	0.19 ± 0.15
Citrus (servings)	0.04 ± 0.02	−0.08 ± 0.06
Other fruit (servings)	−0.01 ± 0.03	−0.16 ± 0.13
Total fruit (servings)	−0.00 ± 0.03	−0.23 ± 0.14
Non-whole grain (servings)	0.54 ± 0.11	0.88 ± 0.53
Whole grain (servings)	0.11 ± 0.03	0.08 ± 0.10
Total grains (servings)	0.68 ± 0.11	0.93 ± 0.52
Egg (oz. equivalent)	−0.15 ± 0.05	−0.32 ± 0.25
Red meat (oz.)	0.21 ± 0.04	0.33 ± 0.16
All meat (oz.)	0.43 ± 0.09	0.39 ± 0.34
Animal-sourced food	−0.06 ± 0.17	−0.32 ± 0.8
Potato (servings) *	0.01 ± 0.01	−0.04 ± 0.02
Starchy vegetables (servings)	0.02 ± 0.01	−0.02 ± 0.02
Total vegetables (servings)	0.10 ± 0.02	−0.04 ± 0.09
Starch-rich foods (servings) *	1.1 ± 0.3	4.2 ± 1.6
Sugar-sweetened beverages (g)	0.64 ± 8.73	17.6 ± 40.6
Water (g)	56.26 ± 13.57	111.31 ± 73.02

^1^ Mean and standard error. Significance using a *t*-test: * *p* < 0.05

**Table 4:** Adjusted odds ratios of measures of daily dietary changes with the presence of acanthosis nigricans (AN) at two-year follow-up (n = 558).

Changes in Dietary Measures	Odds Ratio ^1^	Confidence Interval
Energy (kcal)	1.00	1.00–1.00
Carbohydrates (g)	1.00	1.00–1.00
Protein (g)	1.00	0.98–1.03
Fat (g)	1.00	0.97–1.02
Added sugar (tsp) *	1.09	1.00–1.19
Added fat (g)	0.99	0.94–1.03
Milk (servings)	1.14	0.54–2.41
Total dairy (servings)	0.82	0.40–1.70
Citrus (servings) *	0.07	0.01–0.75
Other fruit (servings)	0.39	0.12–1.28
Total fruit (servings) *	0.33	0.12–0.89
Non-whole grain (servings)	1.07	0.87–1.34
Whole grain (servings)	0.45	0.15–1.31
Total grains (servings)	1.04	0.83–1.31
Egg (oz. equivalent)	1.00	0.66–1.53
Red meat (oz.)	1.29	0.68–2.48
Meat (oz.)	1.02	0.76–1.37
Animal-sourced food	1.00	0.86–1.16
Potato (servings)	0.32	0.00–35.08
Other starchy vegetables (servings)	0.20	0.00–23.69
Total vegetables (servings)	0.66	0.16–2.69
Starch-rich foods (servings) *	1.08	1.00–1.16
Sugar-sweetened beverages (g)	1.00	1.00–1.00
Water (g)	1.00	1.0–1.00

^1^ Adjusted for baseline AN status, age, sex, experimental group, baseline body mass index (BMI) z-score, change in BMI z-score, and baseline intake of dietary measure. A total of 28 children had AN present at follow-up. Significance using multiple logistic regression: * *p* < 0.05.

**Table 5:** Children’s characteristics by follow-up acanthosis nigricans (AN) status.

Measurements	Never AN (n = 508) $\bar{x}\pm \mathrm{SE}$ ^1^ (n)	Chronic AN (n = 12) $\bar{x}\pm \mathrm{SE}$ (n)	Develop AN (n = 22) $\bar{x}\pm \mathrm{SE}$ (n)	AN Remission (n = 16) $\bar{x}\pm \mathrm{SE}$ (n)
Age (months) **	64.0 ± 0.94	80.4 ± 5.68	73.4 ± 3.92	59.8 ± 6.27
Boys %	52.2	83.3	54.6	62.5
Baseline BMI z-score ***	0.42 ± 0.05	2.58 ± 0.16	2.11 ± 0.13	1.34 ± 0.35
2-year change BMI z-scores	0.06 ± 0.03	−0.01 ± 0.12	0.25 ± 0.12	−0.30 ± 0.33

BMI = body mass index. ^1^ Mean and standard error. Significance using ANOVA: ** *p* < 0.01; *** *p* < 0.001.

**Table 6:** Adjusted ^1^ multinominal odds ratios (MOR) of two-year BMI and daily dietary changes and acanthosis nigricans status.

	Chronic MOR (SE) ^2^ (n = 12)	Developed MOR (SE) (n = 22)	Remission MOR (SE) (n = 16)
Energy (kcals)	1.00 (0.00)	1.00 (0.00)	1.00 (0.00)
Carbohydrates (g)	0.99 (0.00)	1.00 (0.00)	1.00 (0.00)
Protein (g)	0.98 (0.02)	1.00 (0.02)	1.00 (0.02)
Fat (g)	0.98 (0.02)	0.98 (0.02)	0.98 (0.03)
Added fat (g)	0.95 (0.03)	1.00 (0.02)	1.01 (0.02)
Added sugar (tsp)	0.80 (0.10) *	1.13 (0.05) **	1.01 (0.05)
Milk (servings)	0.91 (0.55)	1.37 (0.56)	1.35 (0.60)
Dairy (servings)	0.37 (0.24)	0.74 (0.41)	1.09 (044)
Citrus (servings)	0.05 (0.08)	0.09 (0.11)	0.40 (0.43)
Other fruit (servings)	0.53 (0.50)	0.52 (0.34)	1.10 (0.64)
Total fruit (servings)	0.29 (0.23)	0.41 (0.23)	0.69 (0.32)
Non-whole grain (servings)	0.75 (0.15)	1.13 (0.14)	1.02 (0.14)
Total grains (servings)	0.72 (0.12)	1.11 (0.14)	1.06 (0.15)
Whole grain (servings)	0.56 (0.50)	0.35 (0.29)	1.49 (0.77)
Egg (oz. equivalent)	0.69 (0.25)	1.05 (0.26)	0.81 (0.20)
Red meat (oz.)	1.64 (0.94)	0.94 (0.37)	0.52 (0.20)
Meat (oz.)	0.96 (0.22)	1.05 (0.18)	0.98 (0.17)
Animal-sourced food	0.94 (0.10)	1.02 (0.08)	0.98 (0.09)
Potato (servings)	11.68 (43.89)	0.64 (1.64)	0.20 (0.54)
Starchy vegetables (servings)	0.00 (0.02)	0.38 (1.07)	0.03 (1.00)
Total veggies (servings)	0.63 (0.70)	0.61 (0.48)	0.39 (032)
Starch-rich foods (servings)	0.84 (0.05) *	1.12 (0.05) **	1.01 (0.04)
Sugar-sweetened beverages (g)	1.00 (0.00)	1.00 (0.00)	1.00 (0.00)
Water (g)	1.00 (0.00)	1.00 (0.00)	1.00 (0.00)

BMI = body mass index. ^1^ Adjusted each model for age, sex, experimental group, baseline BMI, change in BMI z-score over two years, time between assessments, and baseline food intake. Never group (n = 508). ^2^ Standard error. Significance: * *p* ≤ 0.05; ** *p* ≤ 0.01

## Data Availability

Data described in the manuscript, code book, and analytic code will be made available upon request pending (e.g., application and approval, payment, other).

## Supplementary Materials

The following supporting information can be downloaded at: https://www.mdpi.com/article/10.3390/nu15122718/s1, Figure S1: Consort design—study participants; Table S1: Baseline daily dietary intake of children in four acanthosis nigricans groups; Table S2: Change in dietary intake of children in four acanthosis nigricans groups.
