# Supplementary material for: Diet and Acanthosis Nigricans over a Two-Year Period in Children of the Pacific Region
Source: Nutrients. 2023 Jun 12;15(12):2718. doi: 10.3390/nu15122718 (PMC10302045; doi:10.3390/nu15122718)
Supplement: Supplementary file 1 [file nutrients-15-02718-s001.zip › nutrients-2416369-supplementary.pdf]

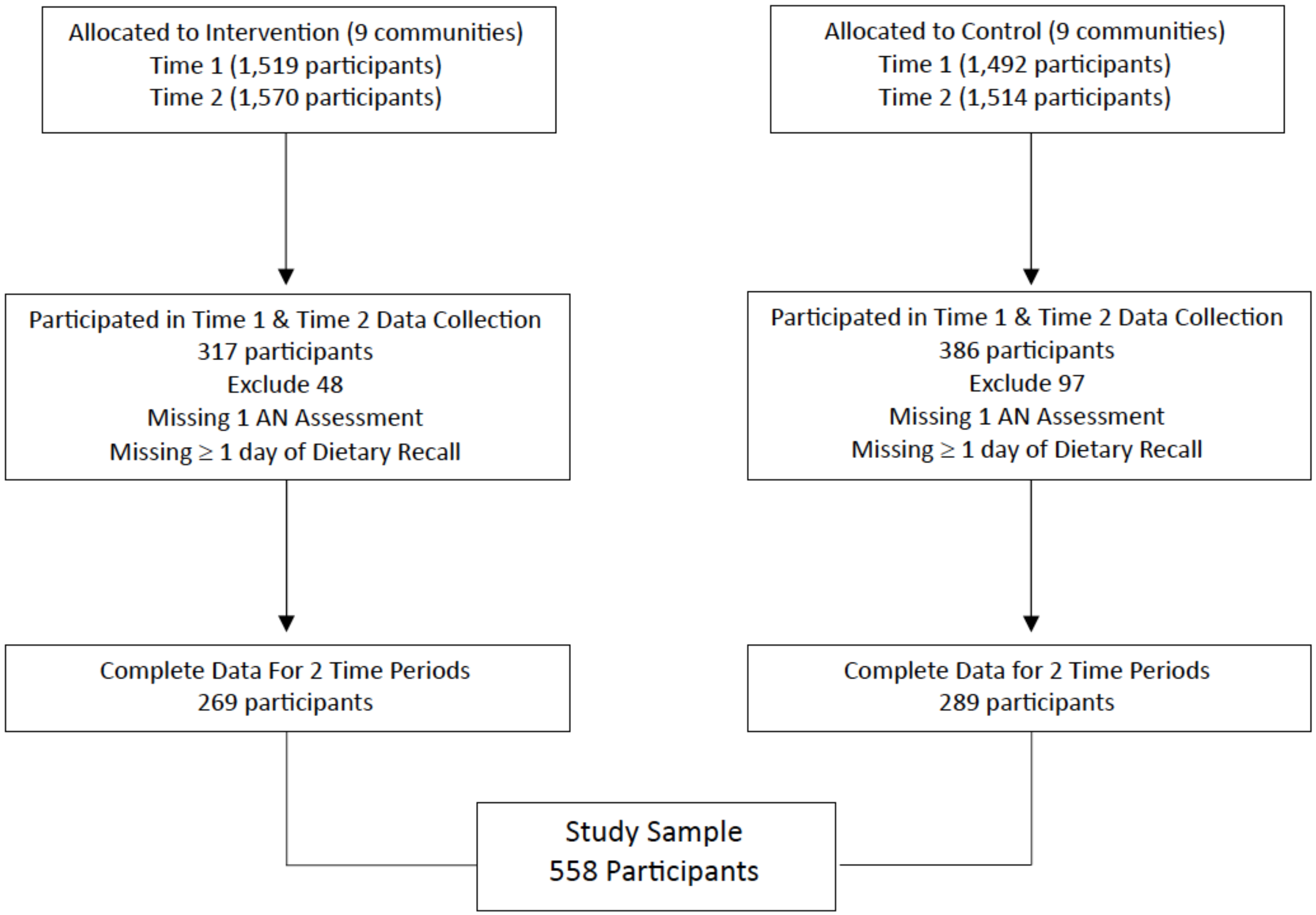

Supplemental Figure S1. Consort Design – Study Participants

Supplemental Table S1. Baseline daily dietary intake of children in four acanthosis nigricans groups

Nutrient/Food Group	Never (n=508) $\bar{x} \pm SE^1$	Chronic (n=12) $\bar{x} \pm SE$	Develop (n=22) $\bar{x} \pm SE$	Remission (n=16) $\bar{x} \pm SE$
Energy (kcal)	1888.9 ± 23.7	2116.3 ± 133.3	1886.1 ± 127.3	1729.9 ± 114.2
Carbohydrate (g)	253.1 ± 3.3	265.7 ± 19.6	239.9 ± 17.3	234.4 ± 16.5
Protein (g)*	69.8 ± 0.9	87.0 ± 5.2	73.6 ± 4.7	66.9 ± 4.3
Fat (g)	66.8 ± 0.9	76.8 ± 4.8	69.0 ± 5.0	59.4 ± 4.6
Added Fat (g)	32.7 ± 0.4	38.8 ± 2.4	32.9 ± 2.5	28.3 ± 1.7
Added Sugar (tsp)	11.6 ± 0.2	9.8 ± 1.7	9.8 ± 0.8	9.3 ± 1.0
Milk	1.2 ± 0.03	1.2 ± 0.2	1.0 ± 0.1	1.1 ± 0.1
Total Dairy	1.4 ± 0.0	1.5 ± 0.1	1.3 ± 0.1	1.3 ± 0.1
Citrus (servings)	0.27 ± 0.01	0.33 ± 0.07	0.26 ± 0.05	0.22 ± 0.06
Other Fruit (servings)	0.80 ± 0.02	0.92 ± 0.18	0.66 ± 0.08	0.70 ± 0.13
Total Fruit (servings)	1.15 ± 0.03	1.34 ± 0.23	0.95 ± 0.10	0.99 ± 0.17
Non-Whole Grain (servings)	6.46 ± 0.10	7.26 ± 0.58	6.77 ± 0.53	6.41 ± 0.52
Whole Grains (servings)	0.38 ± 0.02	0.31 ± 0.11	0.45 ± 0.09	0.33 ± 0.12
Total Grain (servings)	7.01 ± 0.10	7.75 ± 0.57	7.38 ± 0.55	6.97 ± 0.50
Egg (oz equivalent)	0.49 ± 0.02	0.44 ± 0.12	0.60 ± 0.08	0.51 ± 0.09
Red Meat (oz)**	0.78 ± 0.03	1.30 ± 0.27	0.81 ± 0.14	0.79 ±0.14
All Meat	4.67 ± 0.08	6.51 ± 0.52	5.21 ± 0.40	4.33 ± 0.34
Animal Sourced Food*	6.98 ± 0.15	8.74 ± 0.98	7.65 ± 0.84	6.26 ± 0.71
Potato (servings)*	0.08 ± 0.00	0.12 ± 0.04	0.14 ± 0.02	0.13 ± 0.04
Starchy Veggies (servings)	0.07 ± 0.01	0.09 ± 0.02	0.11 ± 0.02	0.04 ± 0.02
Total Vegetables (servings)	0.7 ± 0.02	0.9 ± 0.08	0.8 ± 0.08	0.7 ± 0.10
Starch Rich Foods (servings)	18.2 ± 0.30	17.3 ± 2.2	16.8 ± 1.2	15.9 ± 1.3
Sugar Sweetened Beverages (g)	187.4 ± 7.4	182.3± 59.0	153.0 ± 25.4	101.4 ± 31.8
Water (g)	354.6 ± 10.8	474.7 ± 101.0	401.9 ± 61.6	392.4 ± 53.4

[1] Mean and Standard Error; * $p \le 0.05$; ** $p \le 0.01$

Supplemental Table S2. Change in dietary intake of children in four acanthosis nigricans groups

Nutrient/Food Group	Never (n=508) $\bar{x} \pm$ SE	Chronic (n=12) $\bar{x} \pm$ SE	Develop (n=22) $\bar{x} \pm$ SE	Remission (n=16) $\bar{x} \pm$ SE
Energy (kcal)*	130.87 ± 25.60	-124.75 ± 204.71	429.65 ± 161.22	303.47 ± 150.36
Carbohydrate (g)**	16.20 ± 3.77	-31.35 ± 27.41	72.11 ± 23.03	37.65 ± 26.09
Protein (g)*	6.01 ± 0.96	-1.73 ± 8.05	10.98 ± 5.37	9.82 ± 4.74
Fat (g)	4.72 ± 1.08	0.36 ± 8.32	11.83 ± 6.28	10.99 ± 5.04
Added Fat (g)	2.82 ± 0.54	-1.67 ± 4.16	6.70 ± 3.65	6.36 ± 2.40
Added Sugar (tsp) ***	0.50 ± 0.30	-1.92 ± 1.39	6.31 ± 1.64	2.10 ± 1.47
Milk	0.04 ± 0.03	0.07 ± 0.26	0.25 ± 0.17	0.14 ± 0.21
Total Dairy	0.06 ± 0.04	-0.02 ± 0.26	0.30 ± 0.18	0.12 ± 0.22
Citrus (servings)	0.04 ± 0.02	-0.15 ± 0.07	-0.04 ± 0.08	0.03 ± 0.08
Other Fruit (servings)	-0.01 ± 0.03	-0.30 ± 0.28	-0.08 ± 0.12	0.02 ± 0.21
Total Fruit (servings)	-0.00 ± 0.03	-0.47 ± 0.32	-0.09 ± 0.14	-0.03 ± 0.27
Non-Whole Grain (servings)	0.54 ± 0.11	-0.57 ± 0.72	1.68 ± 0.67	0.68 ± 0.60
Whole Grains (servings)	0.10 ± 0.03	0.25 ± 0.22	-0.02 ± 0.11	0.22 ± 0.18
Total Grain (servings)	0.67 ± 0.11	-0.37 ± 0.70	1.64 ± 0.67	0.94 ± 0.64
Egg (oz equivalent)	-0.14 ± 0.05	-0.78 ± 0.43	-0.06 ± 0.32	-0.37 ± 0.29
Red Meat (oz)	0.22 ± 0.04	0.35 ± 0.35	0.32 ± 0.16	-0.05 ± 0.22
All Meat	0.41 ± 0.09	0.07 ± 0.44	0.56 ± 0.48	0.98 ± 0.51
Animal Sourced Food	-0.09 ± 0.18	-1.28 ± 1.36	0.20 ± 0.98	0.80 ± 1.00
Potato (servings)*	0.01 ± 0.01	-0.02 ± 0.04	-0.06 ± 0.03	-0.06 ± 0.05
Starchy Veggies (servings)	0.02 ± 0.01	-0.02 ± 0.04	-0.02 ± 0.04	-0.02 ± 0.03
Total Vegetables (servings)	0.11 ± 0.02	-0.02 ± 0.12	-0.04 ± 0.12	0.01 ± 0.11
Starch Rich Foods (servings)***	1.07 ± 0.34	-2.53 ± 1.92	7.92 ± 2.00	2.74 ± 1.93
Sugar Sweetened Beverages (g)*	-0.54 ± 8.96	-125.0 ± 69.5	95.45 ± 42.47	37.93 ± 26.87
Water	55.93 ± 13.84	100.29 ± 140.68	117.31 ± 85.37	66.92 ± 67.60

[1] Mean and Standard Error; * $p \leq 0.05$; ** $p \leq 0.01$; *** $p \leq 0.001$
